# Population-Based Study on the All-Cause and Cause-Specific Risks of Mortality among Long-Term Opioid Analgesics Users without Cancer in Taiwan

**DOI:** 10.3390/healthcare9111402

**Published:** 2021-10-20

**Authors:** Po-Feng Lee, Chung-Yi Li, Yen-Chin Liu, Chang-Ta Chiu, Wen-Hsuan Hou

**Affiliations:** 1 Department of Public Health, College of Medicine, National Cheng Kung University, Tainan 704, Taiwan; adusk0910@gmail.com (P.-F.L.); cyli99@mail.ncku.edu.tw (C.-Y.L.); 2 Jianan Psychiatric Center, Ministry of Health and Welfare, Tainan 717, Taiwan; 3 Department of Public Health, College of Public Health, China Medical University, Taichung 406, Taiwan; 4 Department of Anesthesiology, School of Post-Baccalaureate, College of Medicine, Kaohsiung Medical University, Kaohsiung 807, Taiwan; anesliu@kmu.edu.tw; 5 Department of Anesthesiology, College of Medicine, National Cheng Kung University, Tainan 704, Taiwan; 6 Department of Dentistry, An Nan Hospital, China Medical University, Tainan 709, Taiwan; chiouchangta@yahoo.com.tw; 7 Department of Physical Medicine and Rehabilitation, Taipei Medical University Hospital, Taipei 110, Taiwan; 8 School of Gerontology Health Management & Master Program in Long-Term Care, College of Nursing, Taipei Medical University, Taipei 110, Taiwan; 9 Graduate Institute of Clinical Medicine, College of Medicine, Taipei Medical University, Taipei 110, Taiwan

**Keywords:** prescription opioids, mortality, standardized mortality ratio, underlying cause of death

## Abstract

(1) Background: The prevalence of opioid use in Taiwan increased by 41% between 2002 and 2014. However, little is known regarding the risk of mortality among long-term opioid analgesics users who do not have cancer. This study investigated this mortality risk with an emphasis on the calendar year and patients’ age and sex. (2) Methods: This retrospective cohort study included 12,990 adult individuals without cancer who were long-term users of opioid analgesics and were randomly selected from the data set of Taiwan’s National Health Insurance program from 2000 to 2012. They were then followed up through 2013. Information on the underlying causes of death was retrieved from the Taiwan Death Registry. Age, sex, and calendar year-standardized mortality ratios (SMRs) of all-cause and cause-specific mortality were calculated with reference to those of the general population. (3) Results: With up to 14 years of follow-up, 558 individuals had all-cause mortality in 48,020 person-years (cumulative mortality: 4.3%, mortality rate: 11.62 per 1000 person-years). Compared with the general population, the all-cause SMR of 4.30 (95% confidence interval (95% CI): 3.95–4.66) was significantly higher: it was higher in men than in women, declined with calendar year and age, and was significantly higher for both natural (4.15, 95% CI: 3.78–4.53) and unnatural (5.04, 95% CI: 3.88–6.45) causes. (4) Conclusions: Long-term opioid analgesics use among individuals without cancer in Taiwan was associated with a significantly increased risk of mortality. The notably increased mortality in younger adults warrants attention. Strategies to reduce long-term opioid analgesics use, especially their overuse or misuse, are in an urgent need.

## 1. Introduction

Over the past 20 years, the consumption of opioid analgesics has significantly increased in many North American and European countries. Overwhelming international concern has arisen regarding the increase in opioid analgesics addiction and black marketing as well as in opioid intoxication and mortality. Taiwan is no exception. From 2002 to 2007, opioid consumption in Taiwan increased by 55% from 362 to 560 defined daily doses per million inhabitants per day; Taiwan thus ranked 56th out of 181 countries and regions worldwide in opioid consumption, according to the 2005–2007 data set of the International Narcotics Control Board [1]. This trend continued to rise despite the Taiwanese government implementing strict guidelines on the prescription of opioid analgesics. For example, opioid consumption still increased by 41% between 2002 and 2014 [2]. The potential adverse health impacts of the long-term use of opioid analgesics are of both clinical and public health importance due to the increase in opioid consumption.

Both the prevalence and health risks associated with opioid analgesics use have been well documented in the literature. Several studies have compared different countries’ trends in consuming opioid analgesics [3,4,5]. Furthermore, some studies have investigated the mortality risks associated with the use of different opioid analgesics in different populations [6,7,8,9]. However, the trends of all-cause and cause-specific mortality have revealed substantial heterogeneity among nations, calendar years, target populations, and types of opioid analgesics consumed [10,11,12]. A recent meta-analysis of 10 cohorts reported a pooled all-cause crude mortality rate of 28.8 per 1000 person-years (95% confidence interval (95% CI): 17.9–46.4) with substantial heterogeneity (I^2^ = 99.9%) [13]. However, another recent meta-analysis of 16 cohorts estimated a pooled all-cause crude mortality rate of 1.24 per 100 person-years (95% CI: 0.86−1.78) for people with regular or problematic cocaine use; the study also revealed considerable heterogeneity (I^2^ = 98.8%). [14] Moreover, researchers have rarely used age and sex as stratifications when investigating the effects of long-term opioid analgesics use on mortality [13,14].

The potentially increased risk of mortality among the increasing number of individuals without cancer who are long-term users of opioid analgesics in Taiwan has not received adequate attention. Additionally, information regarding the mortality risk associated with consuming opioid analgesics largely originates from Western societies, and little is known of this relationship in Asian populations. This study therefore investigated the all-cause and cause-specific risks of mortality associated with long-term opioid analgesics consumption among individuals without cancer in Taiwan. The risk of all-cause mortality was further stratified according to calendar year, age, and sex.

## 2. Materials and Methods

This study was approved by the Institutional Review Board of Jianan Psychiatric Center, Taiwan Ministry of Health and Welfare (No. 16-007). The requirement of written informed consent was waived due to the deidentification of all data. Data management and all analyses were performed onsite at the Health and Welfare Data Science Center of the Taiwan Ministry of Health and Welfare.

The authors assert that all procedures contributing to this work comply with the ethical standards of the relevant national and institutional committees on human experimentation and with the Helsinki Declaration of 1975, as revised in 2008.

### 2.1. Data Sources

The data analyzed in this study were retrieved from data sets of the National Health Insurance (NHI) program and the Taiwan Death Registry (TDR) from 2000 to 2013. The NHI data sets contain the records of all of Taiwan’s inpatient/outpatient medical claims and the drugs prescribed for treatment, and the National Health Insurance Administration performs a quarterly expert review of a random sample of medical claims to ensure the claims’ accuracy [15]. Additionally, the TDR is considered to be accurate and complete because all deceased residents of Taiwan must be registered, and physicians must provide all patient information on the death certificate, including the patient’s demographic characteristics, underlying cause of death (UCOD), place of death, and marital status [16].

This study used a randomly selected sample of 2 million beneficiaries who were registered in the NHI in 2000. NHI claims and TDR information of this sample between 2000 and 2013 were retrieved and analyzed. This random sample was verified by the Department of Statistics of Taiwan’s Ministry of Health and Welfare for its preventiveness of all Taiwanese residents with respect to age, sex, and geographical distribution of residence [15].

### 2.2. Study Cohort and End Points

The NHI claims revealed that between 2001 and 2012, 92,615 adults received opioid analgesics (i.e., oral morphine, oral fentanyl, oral codeine, oral tramadol, transdermal morphine, or transdermal fentanyl) as either a single prescription for >14 days or a cumulative prescription for >28 days in a 90-day period. We excluded the following users of opioid analgesics: (1) 62,731 users who had cancer-related diagnoses (International Classification of Diseases, Ninth Revision, Clinical Modification (ICD-9-CM)codes: 140–239) in 2000–2013; (2) 16,783 users who were aged <18 or >65 years when they were first prescribed the drugs; (3) 68 users who either had been prescribed opioid analgesics or had received opioid-related diagnoses (ICD-9-CM codes: 292, 305.51–305.53, 304.0, 304.7, 304.9, 965.0, E935.0, E850.1, E950.0, E980.0, and E935.1-935.2) before 2001; and (4) 43 users who had been prescribed two types of opioid analgesics at the same time. The remaining 12,990 adults comprised the study cohort.

### 2.3. Study Design

The study cohort was linked to the TDR according to the patients’ unique personal identification numbers to identify those who had died by the end of 2013. All patients received at least 1 year of follow-up. The UCODs were classified according to ICD-9-CM (for calendar years 2000–2007) or International Classification of Diseases, Tenth Revision, Clinical Modification (for calendar years 2008–2013) codes. During the 14 years of interest, 558 of the included individuals died, namely 362 men and 196 women.

### 2.4. Statistical Analysis

The person-years observed for each person accumulated from the date of cohort enrollment to either date of death or the last day of 2013. Ages at cohort enrollment were categorized as follows: 18–24, 25–34, 35–44, 45–54, and 55–64 years. The person-years were then categorized according to calendar year, sex, and patient age during follow-up. The study cohort contributed a total of 48,020 person-years during the follow-up period (mean ± standard deviation: 2.81 ± 2.14 years).

We compared opioid analgesics users’ risks for all-cause and cause-specific mortality with those of the general population with comparable sex and age during specific calendar years. The UCODs analyzed in this study included various natural causes of death (i.e., infection, neoplasms, metabolic diseases, hematologic diseases, mental disorders, neurological disorders, circulatory diseases, respiratory diseases, digestive diseases, genitourinary diseases, pregnancy, childbirth or complications during the puerperium, skin or subcutaneous diseases, musculoskeletal diseases, perinatal conditions, congenital malformations or deformities, or symptoms/signs not classified elsewhere), unnatural causes of death (i.e., accidents or violence, suicide, or homicide), and unspecified causes of death. Supplementary Table S1 lists the ICD codes for the UCODs analyzed in this study.

To calculate the expected number of deaths among long-term opioid analgesics users, the annual mortality rates were stratified according to age and sex, with those of the general population of Taiwan serving as a reference. The annual age- and sex-specific population sizes during the study period were derived from the national annual household registration statistics published by Ministry of the Interior of Taiwan (https://pop-proj.ndc.gov.tw/main_en/dataSearch.aspx?uid=78&pid=78, accessed on 31 May 2020). The annual average size of the general population during the study period (i.e., 2001–2013) was 22,881,081. Moreover, we calculated the all-cause and cause-specific standardized mortality ratios (SMRs). The all-cause SMR was further stratified according to the calendar year of cohort enrollment, patient age at cohort enrollment, and patient’s sex. The 95% CI for the SMRs was estimated according to the exact estimation [17]. The UCOD distributions were compared between men and women and between patients of different ages at cohort enrollment. The analysis was performed with SAS (version 9.4; SAS Institute, Cary, NC, USA), and the level of significance was set to α = 0.05.

## 3. Results

Table 1 lists the characteristics of the study cohort (60.25% men vs. 39.75% women). Although most patients were enrolled at the age of 45 years or older (67.09%), 15.19% of patients became long-term opioid analgesics users during young adulthood (<35 years). Codeine was the most commonly used opioid analgesics in patients who enrolled in 2001–2003 (57.7%), but the prevalence decreased thereafter to 5.2% between 2010 and 2012. Tramadol, however, gained prevalence over time, accounting for 88.9% (11,553/12,990) of all opioid analgesics that patients initially used (Supplementary Table S2). By the end of 2013, 558 patients had all-cause mortality over 48,020 person-years, representing a cumulative mortality and mortality rate of 4.3% and 11.62 per 1000 person-years. respectively. The calendar year, age, or sex-specific mortality rates are presented in Table 2 and Supplementary Figure S1.

The study cohort had a significantly higher risk of all-cause mortality than the general population, with an age–sex–calendar SMR of 4.30 (95% CI: 3.95–4.66). Both men and women had significantly increased SMRs (4.56 and 3.89, respectively). Patients of all age stratifications also had significantly increased SMRs. Notably, the youngest group (patients aged 18–24 years) had an even higher SMR (13.17, 95% CI: 8.68–18.58). The age-specific SMRs gradually decreased with increases in age. Enrollment in an earlier calendar year was also significantly associated with a greater SMR; the highest (11.73) and lowest (3.13) SMRs were observed for patients enrolled between 2001 and 2003 and between 2010 and 2012, respectively (Table 2).

Despite the differences in sex-specific and age-specific all-cause SMRs, the UCOD distributions were not significantly different between deceased men and women or across all deceased patients. The deaths of the 85.1% of men and 88.3% of women were attributable to various natural causes (Supplementary Table S3). The leading natural causes of death in men were circulatory disease (*n* = 91), digestive disease (*n* = 75), and metabolic disease (*n* = 37), whereas the leading natural causes of women’s deaths were mainly attributable to metabolic disease (*n* = 42), circulatory disease (*n* = 36), and genitourinary disease (*n* = 19) (not listed in the tables). Unnatural causes of death accounted for 11.6% and 10.7% of the total deaths of men and women, respectively. The discrepancy in UCOD distribution between men and women was not statistically significant (*p* = 0.234).

Supplementary Table S4 presents the age-specific number and proportion of various causes of death. The proportion of natural causes of death (74.1% of patients aged 18–24 years and 89.5% of those aged 55–64 years) tended to be higher among individuals who were older at cohort enrollment. Furthermore, unnatural causes of death and unspecified causes of death were more prevalent in younger adults. Nonetheless, these age-related discrepancies in UCOD distribution had no statistical significance (*p* = 0.519).

Cause-specific analyses revealed that the study cohort had a significantly increased risk of mortality from both natural (SMR = 4.15, 95% CI: 3.78–4.53) and unnatural causes (SMR = 5.04, 95% CI: 3.88–6.45). While the cause of death with the greatest increase in SMR was congenital anomalies (SMR = 58.15, 95% CI: 11.69–139.97), it was based on only three deaths. Such an increased SMR is unreliable and should be interpreted with caution because of a very wide confidence interval. Musculoskeletal and connective tissue diseases (20.88), infections and parasitic diseases (12.93), diseases of the nervous system or sensory organs (11.93), and hematological diseases (10.26) were all associated with greater long-term use of opioid analgesics, with an SMR that was 10 times higher than that of the controls. By contrast, the SMR for cancer was significantly lower among long-term users of opioid analgesics (SMR = 0.29, 95% CI: 0.16–0.47). For unnatural causes, significantly more deaths due to accidents/violence (SMR = 4.52, 95% CI: 3.15–6.14) or suicide (SMR = 5.88, 95% CI: 3.91–8.25) were observed in people without cancer who were long-term users of opioid analgesics (Table 3).

## 4. Discussion

This study identified a relatively high all-cause SMR in 12,990 individuals without cancer who were long-term users of opioid analgesics in Taiwan, both among individuals with all-cause mortality across different calendar year, age, and sex stratifications as well as among individuals who died of natural and unnatural causes. To the best of our knowledge, this study is the first of its kind with an Asian cohort, and the results are comparable to the findings presented in studies with Western cohorts. Global opioid consumption increased substantially after the year 2000, disproportionately so in high-income countries, with severe consequences for mortality and morbidity. Codeine remains the most commonly used opioid analgesic, but stronger opioids, such as oxycodone, are becoming more common [18]. In contrast to international statistics on tramadol use, tramadol has been the most common opioid analgesic used long term by individuals without cancer in Taiwan.

Based on 10 cohorts, Larney et al. estimated a pooled all-cause crude mortality rate of 28.8 per 1000 person-years for people who were prescribed opioids, but their estimations exhibited substantial heterogeneity not only between countries, but also within countries [13]. The lowest and highest all-cause crude mortality rates were reported by Foster et al. in the United States (8.95 per 1000 person-years) [19] and Du et al. in Germany (57.70 per 1000 person-years), respectively [20]. More recently, Peacock et al. reviewed 16 cohort studies and reported a pooled all-cause crude mortality rate of 12.4 per 1000 person-years among people with regular or problematic cocaine use [14]. Similar to the findings of Larney et al. [13], Peacock et al. also indicated considerable geographic variations in all-cause crude mortality rate, with the highest figure being noted for studies conducted in tropical Latin America (22.8 per 1000 person-years), followed by studies from high-income North American countries (15.6 per 1000 person-years) and Western European countries (9.3 per 1000 person-years) [14]. Based on 92 papers with 101 cohorts (*n* = 101~229,274) that measured all-cause mortality and opioid overdose-specific mortality in North America, Australia, several Eastern and Western European countries, and Asia, Bahji et al. found the overall all-cause mortality rate was 18.7 per 1000 PY (95% CI: 17.1–20.3). The overall overdose-specific mortality rate was 7.0 per 1000 PY (95% CI: 6.1–8.0). All-cause and overdose-specific mortality were substantially higher in low/middle-income countries, among those with HIV, and among people who use injection drugs [21]. Over 48,020 person-years, 558 all-cause deaths were observed our study cohort (the mortality rate, representing an all-cause mortality of 11.62 per 1000 person-years, is comparable to international figures).

By using 16 cohorts and analyzing a total of 69,932 people with regular or problematic cocaine use, Peacock et al. obtained a pooled all-cause SMR of 6.13, with apparent sex (men/women: 3.42/4.59, respectively), age (<30 years/30 years: 7.75/3.09, respectively), and regional heterogeneity (tropical Latin American/Western European/high-income North American countries: 14.75/6.01/5.13, respectively) differences [14]. Based on 43 cohorts, Larney et al. estimated all-cause and cause-specific mortality among people using extra-medical opioids and found a pooled all-cause SMR of 10.0 (95% CI: 7.6–13.2). Excess mortality was observed across a range of causes, including overdose, injuries, and infectious and noncommunicable diseases [22]. Although our study obtained similar results, we also noted a decline in SMR over time, which was likely due to a shorter follow-up period for patients who were enrolled in relatively recent years. In fact, there are no data available suggesting a period most relevant to address the association of opioids with mortality. Among the 13 cohort studies included in the systematic review and meta-analysis of all-cause and overdose mortality risk among people prescribed opioids [13], only 2 studies followed study participants for at least 1 year, 1 study set a follow-up period of at least 5 years, and the others did not set any minimum time period required for follow-up. Moreover, Dart et al. described trends in the diversion and abuse of prescription opioid analgesics in the US between 2002 and 2013 and found that prescriptions for opioid analgesics increased substantially from 2002 through 2010 in the US but then decreased slightly from 2011 through 2013. The rate of opioid-related deaths rose and fell in a similar pattern, suggesting no obvious lag between opioid use and mortality [23].

Nonetheless, researchers should proceed with caution when interpreting the relatively increased SMRs because of the potential of confounding by indication, wherein the underlying medical conditions of users of opioids may also influence mortality. To address this potential methodological problem, Tölle et al. included four studies with seven study arms and 120,186 patients, and they calculated a pooled covariate adjusted hazard ratio (aHR) of 1.69 (95% CI: 1.47–1.95) for all-cause mortality [24]. When they confined mortality risk to out-of-hospital deaths, they obtained a pooled aHR of 2.12 (95% CI: 1.46–3.09) [24]. Moreover, the use of opioid analgesics is typically accompanied by the use of other pain relievers, such as nonsteroidal anti-inflammatory drugs, which makes the specific association of opioid analgesics with mortality difficult to evaluate. Although comparisons of SMRs across studies have potential problems [25], our study results were generally comparable to the findings of other research studies.

In the aforementioned studies by Peacock et al. and Tölle et al., congenital anomalies and hematological diseases exhibited a more than tenfold increase in SMR; however, both studies were based on a relatively small number of deaths. The increase in mortality from natural causes was associated with musculoskeletal and connective tissue diseases, infection and parasitic diseases, and diseases of the nervous system and sensory organs. Musculoskeletal pain is pain that affects bones, joints, ligaments, muscles, and tendons and is prevalent in both middle-aged and older adults. Chronic pain resulting from musculoskeletal and connective tissue diseases is one of the leading causes of disability [26], which might in turn increase the risk of mortality. The increased SMR for neurological diseases may be attributable to certain neuroplastic events within the mesocorticolimbic system that emerge due to chronic exposure to opioids. It may have a determinative influence on behavioral symptoms associated with opioid use disorder, which is a chronic relapsing clinical condition with remarkably high morbidity and mortality [27]. The remarkably low SMR for neoplasm in the present is due to the study cohort being restricted to people without cancer.

In our study, suicide was an unnatural cause of death that had one of the most elevated SMRs, which aligns with previous findings that patients with chronic pain are at an increased risk of suicide [28,29]. Many factors promote the initiation and persistence of opioid use, but the pathways toward vulnerability to overdose and suicide are related to biological, medical, and social factors [30]. A recent meta-analysis also reported increased SMRs for suicide (SMR: 7.93, 95% CI: 5.69–11.04), unintentional injury (SMR: 6.85, 95% CI: 4.41–10.64), and violence (SMR: 9.75, 95% CI: 6.60–14.39) [22]. Although our study also observed increased SMRs (4.52) for accidents and violence, no deaths due to homicide were observed in our study cohort.

Opioid use disorders affect over 16 million people worldwide, including over 2.1 million in the United States, and over 120,000 deaths worldwide annually are attributed to opioid use [31]. Although the rates of misuse of prescription medicine, including opioids, have been reported to be lower in countries in the Asia–Pacific region than in many Western countries (such as the United States and United Kingdom), adolescents and young adults in Asia–Pacific and Western countries exhibit similar trends of misuse. The problems with misuse in the Asia–Pacific region could be overlooked because the association between drug misuse and health consequences, such as mortality, are not well documented by most countries in the region [32].

Chronic pain is one of the most common symptoms reported by patients in outpatient clinics. However, failure to manage chronic pain and opioid dependence associated with chronic pain can result in high rates of morbidity and mortality. Moreover, pain-related expenses are extremely high and represent a substantial burden [26]. Besides, interventions such as marijuana laws, harm-reduction interventions, health insurer policies, and patient/health care provider education, as well as simultaneous interventions on opioid-related outcomes, have also been used to reduce the inappropriate prescription of drugs [33]. Some patients with chronic pain are treated with opioid analgesics regularly for pain relief, and likely to become long-term opioid analgesics users. Awareness and health literacy regarding the potential adverse effect from opioid use should be enhanced by shared decision making, a process by which the clinician and the patient share all applicable information and negotiate a plan of pain treatment that is acceptable to both [34].

Although this study used a population-based approach with a large number of unselected study participants, which minimized the likelihood of selection bias and allowed for analyses of mortality from specific causes, several limitations should be noted. First, we were unable to differentiate between misuse/overuse and appropriate use of opioids. Second, medical claims do not cover the information of extra-medical opioid use, which could entail certain degrees of exposure misclassification and could likely underestimate the association between long-term opioid analgesics use and mortality. Third, despite that an elevated risk of mortality was found in long-term opioid analgesics users, we did not weigh the risks and benefits of long-term opioid analgesics use. After all, pain control by medications is essential in assuring the quality of life in patients with chronic pain.

## 5. Conclusions

In conclusion, long-term opioid analgesics use among individuals without cancer in Taiwan was associated with a significantly increased risk of mortality. The notably increased mortality in younger adults warrants attention. Strategies to reduce long-term opioid analgesics use, especially their overuse or misuse, are urgently needed.

## Figures and Tables

**Table 1:** Characteristics of the study cohort.

Characteristics	*n*	%
Total	12,990	100.00
Calendar year of enrollment ^a^		
2001–2003	360	2.77
2004–2006	789	6.07
2007–2009	3798	29.24
2010–2012	7843	60.38
Age at cohort enrollment (years)		
18–24	474	3.65
25–34	1499	11.54
35–44	2302	17.72
45–54	3682	28.34
55–64	5033	38.75
Mean ± SD	48.52 ± 11.63	
Sex		
Male	7826	60.25
Female	5164	39.75
Years of follow-up		
<2	5445	41.92
2–3	3878	29.85
4–5	2301	17.71
6–7	713	5.49
8–9	300	2.31
10–14	353	2.72
Mean ± SD	2.81 ± 2.14	
Survival status at the end of 2013		
Survivors	12,432	71.42
Nonsurvivors	558	28.58

^a^ Based on the date of the first inpatient/outpatient visit with opioid analgesics usage between 2000 and 2013. Abbreviation: SD, standard deviation.

**Table 2:** All-cause standardized mortality ratios among individuals without cancer who were long-term users of opioid analgesics.

All-Cause Mortality	Obs.	Mortality Rate (per 10^3^ Person-Years)	Exp.	Standardized Mortality Ratio ^a^		
				Estimate	95% CI	
Overall	558	11.62	129.77	4.30	3.95	4.66
By calendar year of enrollment						
2001–2003	23	16.74	1.96	11.73	7.44	17.61
2004–2006	67	14.31	8.17	8.20	6.36	10.41
2007–2009	132	9.84	23.54	5.61	4.69	6.65
2010–2012	336	10.90	96.10	3.50	3.13	3.89
By age at cohort enrollment (years)						
18–24	27	1.77	2.05	13.17	8.68	18.58
25–34	94	6.16	14.33	6.56	5.30	7.95
35–44	93	8.31	18.36	5.07	4.09	6.15
45–54	144	11.56	31.07	4.63	3.91	5.42
55–64	200	15.92	63.96	3.13	2.71	3.57
By sex						
Men	362	13.40	79.85	4.56	4.10	5.04
Women	196	9.14	49.92	3.89	3.36	4.45

^a^ Standardized for sex, age, and calendar year. Abbreviations: Obs., observed number; Exp., expected number; CI, confidence interval.

**Table 3:** Cause-specific standardized mortality ratios in individuals without cancer who were long-term users of opioid analgesics.

Underlying Cause of Death	Obs.	Mortality Rate (103 Person-Years)	Exp.	Standardized Mortality Ratio ^a^		
				Estimate	95% CI	
Natural causes of death	481	10.02	116.30	4.15	3.78	4.53
Infection and parasitic diseases	29	0.60	2.24	12.93	8.66	18.05
Neoplasms ^b^	13	0.27	44.27	0.29	0.16	0.47
Metabolic and immunity diseases	79	1.65	10.27	7.69	6.09	9.48
Hematological diseases	3	0.06	0.29	10.26	2.06	24.70
Mental disorders	2	0.04	0.64	3.14	0.35	8.74
Diseases of the nervous system and sensory organs	16	0.33	1.34	11.93	6.81	18.45
Circulatory diseases	127	2.65	26.62	4.77	3.98	5.64
Respiratory disease	42	0.87	9.52	4.41	3.18	5.84
Digestive diseases	88	1.83	10.67	8.25	6.61	10.06
Genitourinary disease	47	0.98	5.98	7.85	5.77	10.25
Complications of pregnancy, childbirth, and the puerperium	0	0.00	0.00	NA		
Skin and subcutaneous disease	3	0.06	0.67	4.45	0.89	10.71
Musculoskeletal and connective tissue diseases	13	0.27	0.62	20.88	11.11	33.68
Congenital anomalies	3	0.06	0.05	58.15	11.69	139.97
Conditions originating in the perinatal period	0	0.00	0.00	NA		
Symptoms/signs not classified elsewhere	16	0.33	3.12	5.12	2.93	7.92
Unnatural causes of death	63	1.31	12.49	5.04	3.88	6.45
Accidents and violence	35	0.73	7.73	4.52	3.15	6.14
Suicide	28	0.58	4.76	5.88	3.91	8.25
Homicide	0	0.00	0.18	NA		
Unspecified causes of death	14	0.29	0.77	18.25	9.97	28.99

Abbreviations: Obs., observed number; Exp., expected number; CI, confidence interval; NA, not applicable due to limited number of deaths. ^a^ Standardized for sex, age, and calendar year, ^b^ These deceased cancer patients were not present in NHI claims during the follow-up period.

## Data Availability

Data management and all analyses were performed onsite at the Health and Welfare Data Science Center of the Taiwan Ministry of Health and Welfare. Data is not available to the public and data sharing is prohibited under the current government regulations.

## Supplementary Materials

The following are available online at https://www.mdpi.com/article/10.3390/healthcare9111402/s1, Supplementary Table S1: International Classification of Diseases’ codes for the diseases analyzed in this study; Supplementary Table S2: Comparison of opioid analgesics consumed by patients enrolled in different calendar years; Supplementary Table S3: Comparison of underlying causes of death between male and female users of long-term opioid analgesics; Supplementary Table S4: Comparison of underlying causes of death among long-term users of opioid analgesics with respect to their age at cohort enrollment; Supplementary Figure S1: Mortality rate according to calendar year of enrollment (upper), age (years) at cohort enrollment (middle), or sex (lower).
