# Supplementary material for: Population-Based Study on the All-Cause and Cause-Specific Risks of Mortality among Long-Term Opioid Analgesics Users without Cancer in Taiwan
Source: Healthcare (Basel). 2021 Oct 20;9(11):1402. doi: 10.3390/healthcare9111402 (PMC8625753; doi:10.3390/healthcare9111402)
Supplement: Supplementary file 1 [file healthcare-09-01402-s001.zip › healthcare-1394626-supplementary.pdf]

Supplementary Table S1. International Classification of Diseases codes for the diseases analyzed in this study

Diseases	ICD-9-CM	ICD-10-CM
Natural causes of death		
Infection and parasitic diseases	001–139	A010–B99
Neoplasms	140–239	C00-D48
Metabolic and immunity diseases	249–279	E00–E90
Hematological diseases	280–289	D50–D89
Mental disorders	290–319	F00–F99
Diseases of the nervous system and sensory organs	320–389	G00–G99
Circulatory diseases	390–459	I00–I99
Respiratory diseases	460–519	J00–J99
Digestive diseases	520–579	K00–K93
Genitourinary diseases	580–629	N00–N99
Complications during pregnancy, childbirth, or the puerperium	630–676	O00–O99
Skin and subcutaneous tissue diseases	680–709	L00–L99
Musculoskeletal and connective tissue diseases	710–739	M00–M99
Congenital anomalies	740–759	Q00–Q99
Conditions originating in the perinatal period	760–799	P96
Symptoms/signs not classified elsewhere	780–799	R00–R99
Unnatural causes of death		
Accidents and violence	E800–E949	V01–X59, Y85–Y86
Suicide	E950-E959	X60–X84, Y87
Homicide	E960–E969	X85–Y09, Y87.1
Unspecified cause of death	NA	NA

Abbreviations: NA, not applicable; ICD-9-CM, International Classification of Diseases, Ninth Revision, Clinical Modification; ICD-10-CM, International Classification of Diseases, Tenth Revision, Clinical Modification

Supplementary Table S2. Comparison of opioid analgesics consumed by patients enrolled in different calendar years

Opioid analgesics	Total	2001–2003		2004–2006		2007–2009		2010–2012	
		n	%	*n*	%	*n*	%	*n*	%
Fentanyl	14	4	0.7	4	0.5	3	0.1	3	0.0
Morphine	33	4	0.7	10	1.3	14	0.4	5	0.1
Tramadol	11,553	229	40.9	423	53.6	3473	91.4	7428	94.7
Codeine	1390	323	57.7	352	44.6	308	8.1	407	5.2
	12,990	560	100.0	789	100.0	3798	100.0	7843	100.0

Supplementary Table S3. Comparison of underlying causes of death between male and female users of long-term opioid analgesics

Underlying cause of death	Males		Females		p value [a]
	n	%	*n*	%	
Natural causes	308	85.1	173	88.3.	0.234
Unnatural causes	42	11.6	21	10.7	
Unspecified causes	12	3.3	2	1.0	
Total	362	100.0	196	100.0	

[a] Based on a χ^2 test

Supplementary Table S4. Comparison of underlying causes of death among long-term users of opioid analgesics with respect to their age at cohort enrollment

Underlying cause of death	18–24 years		25–34 years		35–44 years		45–54 years		55–64 years		*p* value
	n	%	*n*	%	*n*	%	*n*	%	*n*	%	
Natural causes	20	74.1	78	83.0	80	86.0	124	86.1	179	89.5	0.519
Unnatural causes	5	18.5	13	13.8	11	11.8	17	11.8	17	8.5	
Unspecified causes	2	7.4	3	3.2	2	2.2	3	2.1	4	2.0	
Total	27	100.0	94	100.0	93	100.0	144	100.0	200	100.0	

[a] Based on a χ^2 test

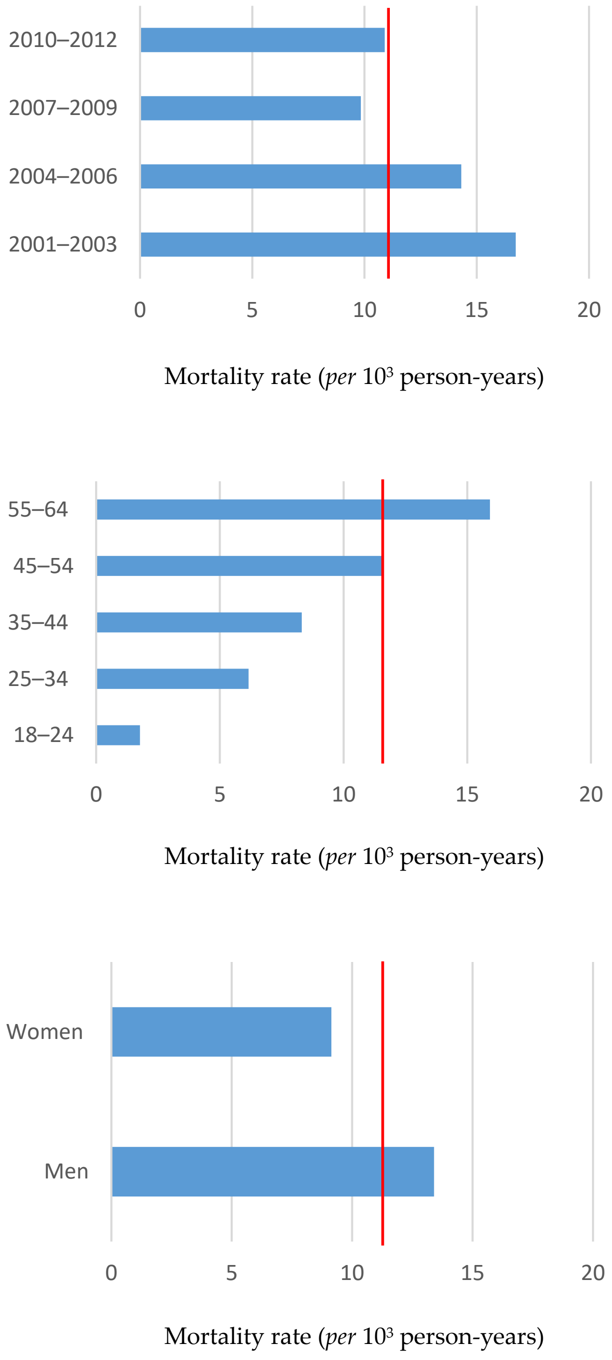

Supplementary Figure S1. Mortality rate according to calendar year of enrollment (upper), age (years) at cohort enrollment (middle), or sex (lower). The red bar indicates the overall mortality (11.62 *per* 10^3 person-years) of all long-term users of opioid analgesics users.
